# Hyperfine-resolved rovibrational and rotational spectroscopy of OH^+^ (*X*^3^Σ^−^)

**DOI:** 10.1039/d6cp00632a

**Published:** 2026-04-28

**Authors:** Weslley G. D. P. Silva, Lea Schneider, Urs U. Graf, Holger S. P. Müller, Pavol Jusko, Arshia M. Jacob, Dominik Riechers, Stephan Schlemmer, Oskar Asvany

**Affiliations:** a I. Physikalisches Institut, Universität zu Köln Zülpicher Str. 77 50937 Köln Germany asvany@ph1.uni-koeln.de; b Max-Planck-Institut für Radioastronomie Auf dem Hügel 69 53121 Bonn Germany

## Abstract

The OH^+^ (*X*^3^Σ^−^) radical cation has been investigated by combining a 4 K 22-pole ion trap apparatus with high-resolution IR and THz radiation sources. Applying different types of action spectroscopic methods, the fundamental vibrational band in the 3 µm range and the spin manifold of the *N* = 1 ← 0 rotational transition around 1 THz have been extended and refined. Additionally, the spin manifold of the *N* = 2 ← 1 rotational transition, scattered around 2 THz, has been measured for the first time with microwave accuracy. Although all hyperfine components of the pure rotational transitions are affected by considerable Zeeman splittings, a simulation of their contours allowed us to extract the field-free center frequencies with high accuracy. A global fit combining rovibrational and pure rotational transitions from the literature with those newly obtained in this work was performed, leading to improvements in the spectroscopic constants of OH^+^, particularly those in the ground vibrational state.

## Abstract 2

The OH^+^ (*X*^3^Σ^−^) radical cation has been investigated by combining a 4 K 22-pole ion trap apparatus with high-resolution IR and THz radiation sources.

## Graphical abstract

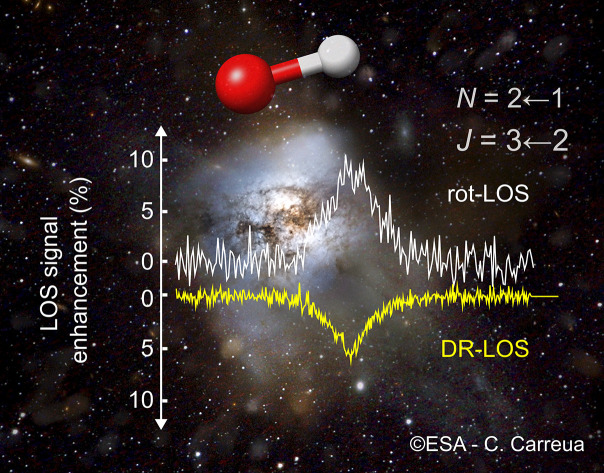

## 1 Introduction

Oxoniumylidene, OH^+^, is a simple radical cation with a *X*^3^Σ^−^ electronic ground state. The first observation of a rotational transition of this molecule was the *N* = 1 ← 0 multiplet which was studied in the laboratory by Bekooy *et al.*^1^ employing laser sideband spectroscopy, with rest frequencies calculated from the *A*^3^Π − *X*^3^Σ^−^ electronic spectrum.^2^ Higher-*N* transitions up to *N* = 3 were studied subsequently by laser magnetic resonance (LMR),^3^ while the *N* = 13 ← 12 rotational transition was recorded by far-IR spectroscopy.^4^ The first high-resolution rovibrational spectrum in the IR was published by Rehfuss *et al.*^5^ with vibrational excitation of hot OH^+^ up to *ν* = 5 ← 4. Müller *et al.*^6^ presented a combined fit of field free data in the ground electronic state^1,4,5^ which was the basis of rest frequencies used to search for OH^+^ in space for a considerable time. Later, using advanced frequency-comb technology, Markus *et al.*^7^ determined more accurate (∼MHz) rest frequencies of the OH^+^*ν* = 1 ← 0 fundamental IR band, which were appended to the combined fit to improve the spectroscopic parameters.

In an astrochemical context, early chemical models predicted that OH^+^ should be abundant in low-density interstellar environments, with its production driven by energetic processes such as interstellar shocks.^8–10^ Tentative observational support for this picture was reported in studies of shocked regions, including early claims of OH^+^ in cometary bow shocks,^11^ although these detections were not definitive. Direct confirmation of widespread interstellar OH^+^ remained challenging for several decades, largely because its ground-state rotational transition lies near 1 THz, a frequency range that is difficult to access from the ground owing to low atmospheric transmission, typically requiring exceptional weather conditions or space-/air-borne facilities.

Unambiguous detection of interstellar OH^+^ was achieved only in 2010 through observations of the hyperfine components of its *N*_j_ = 1_0_ ← 0_1_ transition near 909 GHz^12^ with the APEX telescope. This breakthrough demonstrated that OH^+^ is indeed present in diffuse and translucent regions of the interstellar medium (ISM) and opened the door to systematic studies. Subsequent observations using space- and air-borne platforms, most notably the *Herschel* Space Observatory and SOFIA (Stratospheric Observatory for Infrared Astronomy), enabled access to multiple low-lying rotational transitions and revealed OH^+^ in both absorption and emission across a broad range of interstellar environments.^13–18^ In parallel, OH^+^ was also studied at ultraviolet wavelengths through its *A*^3^Π − *X*^3^Σ^−^ electronic system,^2,19^ providing complementary access to its spectroscopy and abundance in diffuse gas.^20–23^ These measurements established OH^+^ as a widespread constituent of the ISM rather than a molecule confined to extreme or highly localized regions. The close coupling between OH^+^ chemistry and the ionization balance makes OH^+^, especially when considered alongside related species such as H_2_O^+^, a sensitive probe of the cosmic-ray ionization rate in diffuse atomic gas with molecular fractions ≤0.1 (see ref. 14, 15, 24 and 25).

Extragalactic detections of OH^+^ have further demonstrated that its sensitivity to cosmic-ray ionization-driven chemical pathways is not unique to the Milky Way, but persists across a broad range of physical conditions and cosmic epochs, from nearby active and star-forming galaxies to the early universe.^26–31^ At higher redshifts, the OH^+^*N* = 1 ← 0 transitions are shifted into frequency ranges accessible to sensitive (sub-)mm interferometers such as ALMA (Atacama Large Millimeter Array) and NOEMA (Northern Extended Millimeter Array). Following its first detection in the highly redshifted starburst galaxy, HFLS3^30^ with a redshift of *z* = 6.34 (*z* = (*f*_emit_ − *f*_obs_)/*f*_obs_), at least one hyperfine component of OH^+^ has now been detected in more than 20 predominantly strongly lensed starburst galaxies at *z* = 1.8–6.3, enabling constraints on the ionization conditions in these intensely star-forming systems.

There have been fewer observations of higher-*N* OH^+^ transitions thus far, including toward a small number of Galactic planetary nebulae,^32^ and toward the nearby galaxies NGC 4418, Arp 220,^28^ and Markarian 231.^33^ Valuable information on the excitation conditions can be obtained from observing these lines in absorption, emission, or a mix of both (so-called P-Cygni profile). In this context, precise laboratory measurements of pure rotational transitions of OH^+^ at higher frequencies, such as the *N* = 2–1 transition at 2 THz, are needed.

Therefore, we employed a cryogenic ion trap apparatus in combination with the powerful leak-out spectroscopy method (LOS^34^), explained in section 2, to revisit the fundamental vibrational mode of OH^+^ (section 4) in the present study. Subsequently, a double-resonance method has been applied to perform the first field-free laboratory measurement of the *N* = 2 ← 1 rotational transition of OH^+^ (section 5). In addition, data of the *N* = 1 ← 0 rotational transition are presented in section 6. With our novel measurements, sixteen hyperfine components could be extracted from the rotational measurements, and a global fit based on all available data is presented in section 8.

## 2 Experiment

The experiments of this study were carried out using one of Cologne's 22-pole cryogenic ion trapping instruments, COLTRAP.^35^ Every second (1 Hz), a pulse of about twenty thousand OH^+^ ions was generated in a storage ion source *via* electron impact ionization of H_2_O vapor (*E*_e^−^_ ≈ 45 eV). These ions were then extracted from the source, selected in a quadrupole mass spectrometer for *m*/*z* = 17, and injected into the 4 K 22-pole ion trap.^36^ Upon entering the trap, the ions were stopped and thermalized to the cryogenic temperature by collisions with helium buffer gas (*n* ≈ 10^13^ cm^−3^), which was added to the trap continuously. During the trapping time of several hundred milliseconds, the stored OH^+^ ion cloud was irradiated with an IR and/or THz beam. The outcome of this interaction has been probed *via* action spectroscopy by releasing the ion cloud into a second quadrupole mass spectrometer and counting the ions in a high-efficiency ion counter. A spectrum of OH^+^ (rovibrational or rotational) can be recorded by counting the ions as a function of the radiation frequency (IR or THz). The different action spectroscopic methods and radiation sources employed in the present work are described in detail below.

### 2.1 Leak out spectroscopy (LOS)

The rovibrational spectrum of the fundamental stretching vibration of OH^+^ was first measured using the leak out spectroscopy (LOS) method, described in detail by Schmid *et al.*^34^ In the last four years, LOS has developed into a mature technique, with numerous recent applications to astrophysically relevant cations.^37–49^ The trapped ions are excited, in most applications of LOS vibrationally by using an IR beam, and after inelastic collisions with a neutral atomic/molecular partner, in this case Ne, which is pulsed additionally into the trap in a 1 : 3 Ne : He mixture, a fraction of this excitation energy is converted into kinetic energy of both collision partners. The additional kinetic energy gained by the ions allows them to escape from the trap and fly toward the detector, where they are counted as a function of the IR wavenumber to produce the rovibrational spectrum.

### 2.2 Double-resonance rotational spectroscopy (DR-LOS)

Once the rovibrational spectrum was investigated, the pure rotational transitions of OH^+^ were measured using a double-resonance scheme based on LOS, or DR-LOS for short.^37^ This method has been applied to measure the *N* = 2 ← 1 rotational line near 2 THz. Here, the IR excitation is tuned to the resonant frequency of a rovibrational transition of the molecule, which creates a constant rovibrational LOS-signal. This signal is then altered by resonant THz excitation, which can either increase (DR-LOS peak) or decrease (DR-LOS dip) the rovibrational LOS signal. If both transitions (IR and THz) start from the same lower quantum state, a dip is observed in the LOS spectrum, as the addressed quantum state is being de-populated by the THz excitation. In contrast, if the lower state of the IR transition corresponds to the upper state of the THz transition, the LOS signal is amplified (peak), as the THz excitation increases the population of the initial state of the rovibrational transition. In both cases, a change in the number of escaping ions can be measured as a function of the THz frequency, thus generating a rotational spectrum. A more thorough review of double resonance schemes can also be found in ref. 37 and 50, and application examples for the DR-LOS method include HCCCO^+^,^37^ c-C_3_H_2_D^+^,^41^ H_2_CCCH^+^,^40^ HCNH^+^,^45^ C_3_H^+^,^51^ NCCO^+^,^52^ and recently HCN^+^.^53^

### 2.3 Light induced inhibition of complex growth (LIICG)

The rotational measurements of the *N* = 1 ← 0 transition of OH^+^ at 1 THz were carried out using a method called light induced inhibition of complex growth (LIICG^54,55^). With LIICG, the rotational excitation of the molecular ion reduces the three-body rate for attaching a He atom to the ion. Therefore, this method is performed by filling the trap with a larger number density of He gas (∼10^15^ cm^−3^) for the He-attachment to proceed, and by counting the number of product ions OH^+^–He (*m*/*z* = 21) after the trapping period. The resonant transition signal is then detected as a decrease (dip) in the OH^+^–He counts. Before the invention of DR-LOS, LIICG as a generally applicable method has been extensively used for the rotational spectroscopy of astrophysically relevant cations.^55–64^

### 2.4 Radiation sources

The narrow linewidth (<10^−4^ cm^−1^) tunable IR excitation was supplied by a continuous wave optical parametric oscillator (cw-OPO, Toptica, model TOPO) operating in the 2.5–3.5 µm spectral region. The power of the OPO admitted to the trap was on the order of a few tens of mW. The frequency of the IR radiation was measured continuously by a wavemeter/spectrum analyzer (Bristol Instruments, model 771A-MIR), which has an absolute accuracy of 0.2 ppm, *i.e.*, about 0.001 cm^−1^. As the 2 THz radiation source, operating in the range 1.83–2.07 THz, the former local oscillator (LO) of the low frequency array (LFA) of the upGREAT receiver^65^ onboard the SOFIA^66^ air-borne observatory was used. In order to overlay the 2 THz radiation with the IR beam, a 1 mm hole in a plane mirror was used. The details of this setup have been explained elsewhere.^67^ An amplifier-multiplier chain (Virginia Diode Inc., VDI) was applied as the 1 THz source. Both THz sources were driven by a synthesizer locked to a rubidium atomic clock.

## 3 Spectroscopic properties of oxoniumylidene

The OH^+^ radical possesses two unpaired electrons (*S* = 1) and a ^3^Σ^−^ ground electronic state which causes all rotational levels with *N* > 0 to split into three fine structure (FS) levels. The nuclear spin of H (*I* = 1/2) leads to hyperfine structure (HFS) which additionally splits each FS level into two. The rotational spacing is much larger than the FS splitting, which in turn is much larger than the HFS effects, so OH^+^ is best described by Hund's case (b). The rotational angular momentum *N*, the electron spin angular momentum *S* and the nuclear angular momentum *I* are coupled sequentially as *J* = *N* + *S* and *F* = *J* + *I*.

The selection rules are Δ*N* = ±1, Δ*J* = ±1 and Δ*F* = 0, ±1. The most intense allowed transitions are those in which neither the electron spin nor the nuclear spin changes; thus Δ*N* = Δ*J* = Δ*F*. Transitions with change in electron or nuclear spin are also allowed; their intensities can be substantial in comparison to the strongly allowed transitions for low quantum numbers, but are usually negligible for high quantum numbers. Moreover, such transitions usually carry more information on the FS or HFS parameters and are more susceptible to external magnetic fields, such as that of Earth, through the Zeeman effect.

The permanent electric dipole moment of OH^+^ has not been determined experimentally to the best of our knowledge. A ground state value of *µ*_0_ = 2.32 D was estimated based on quantum chemical calculations.^68^ Likewise, a *ν* = 1–0 transition dipole moment of 0.185 D for the fundamental vibration has been reported previously.^69^ It should be pointed out that the transition dipole moment alone is not always sufficient to model the IR intensities, as shown for the *ν* = 1–0 band of CH^+^.^70^

## 4 IR measurements

**Fig. 1:**
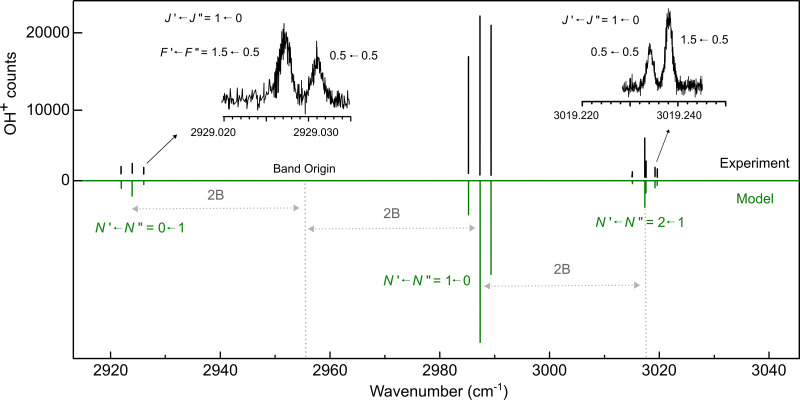
The fundamental vibrational mode of OH^+^ measured with leak-out spectroscopy (LOS). The upper panel (in black) shows the measurement while the lower panel (in green) presents a simulation performed with PGOPHER^71^ at a temperature of 20 K using the fitted spectroscopic parameters from Table 3. The measurement displayed is “single-shot” with no averaging involved. The insets highlight the *N*′ ← *N*″ = 0 ← 1, *J*′ ← *J*″ = 1 ← 0 (left) and *N*′ ← *N*″ = 2 ← 1, *J*′ ← *J*″ = 1 ← 0 (right) rovibrational transitions with their resolvable *F*′ ← *F*″ = 0.5 ← 0.5 and *F*′ ← *F*″ = 1.5 ← 0.5 hyperfine components in detail. Due to the limited number of trapped OH^+^ ions (about 20 000), the strong lines in the *N*′ ← *N*″ = 1 ← 0 branch saturate and therefore, their intensity pattern deviates from the simulation.

A prerequisite for performing double resonance rotational spectroscopy is the knowledge of the rovibrational transitions of the molecule under consideration. Therefore, we revisited the fundamental vibrational transitions of OH^+^ which have been first pioneered by Rehfuss *et al.*^5^ and later refined by frequency-comb-accuracy measurements by Markus *et al.*^7^ We used leak-out spectroscopy (LOS^34^) as the action spectroscopy method in the present study. Our recorded LOS spectrum is shown in Fig. 1. Only one *P*-branch (*N* = 0 ← 1) and two *R*-branch manifolds (*N* = 1 ← 0, *N* = 2 ← 1) could be recorded because of the cryogenic cooling. Also, the low temperature resulted in very narrow Gaussian lines with about 45 MHz FWHM (full width at half maximum). We consider this FWHM to be caused solely by Doppler broadening, corresponding to a kinetic temperature of the OH^+^ ions in the trap of about 12 K, a temperature very typical for our ion trapping experiments.^37,39,60,72^ Other broadening effects appear to be negligible. The observed narrow lines made it possible to resolve some hyperfine components for the first time. Examples are shown in the zoom-in panels given in Fig. 1 and the newly obtained components are tabulated in Table 1.

**Table 1:** Observed transitions of the fundamental vibrational band *ν* = 1 ← 0 of OH^+^ (in cm^−1^). We calibrated our frequencies to the highly accurate lines of Markus *et al.*^7^ which are also given here

*N*′ ← *N*″	*J*′ ← *J*″	*F*′ ← *F*″	This work	Markus *et al.*^7^	obs–calc
0 ← 1	1 ← 1	1.5 ← 1.5, 1.5 ← 0.5	2921.8960 (3)		0.00026
		0.5 ← 0.5, 0.5 ← 1.5	2921.9000 (3)		0.00034
	1 ← 2	1.5 ← 1.5	2923.9369 (3)		−0.00021
		1.5 ← 2.5, 0.5 ← 1.5		2923.940929 (63)	0.000006
	1 ← 0	1.5 ← 0.5	2926.0310 (4)		0.00043
		0.5 ← 0.5	2926.0348 (4)		0.00042
1 ← 0	0 ← 1	0.5 ← 0.5	2985.2294 (4)		−0.00044
		0.5 ← 1.5	2985.2332 (4)		−0.00044
	2 ← 1	2.5 ← 1.5, 1.5 ← 0.5		2987.324274 (96)	0.000008
		1.5 ← 1.5	2987.3280 (1)		−0.00009
	1 ← 1	0.5 ← 0.5, 1.5 ← 0.5	2989.3492 (1)		0.00010
		1.5 ← 1.5, 0.5 ← 1.5		2989.353127 (63)	0.000116
2 ← 1	1 ← 1	0.5 ← 0.5, 0.5 ← 1.5	3015.1026 (2)		−0.00009
		1.5 ← 1.5, 1.5 ← 0.5	3015.1065 (1)		−0.00010
	3 ← 2	3.5 ← 2.5, 2.5 ← 1.5		3017.368784 (61)	0.000024
		2.5 ← 2.5	3017.3726 (1)		−0.00006
	2 ← 1	2.5 ← 1.5, 1.5 ← 0.5, 1.5 ← 1.5		3017.612933 (52)	0.000066
	1 ← 0	0.5 ← 0.5	3019.2375 (1)		0.00009
		1.5 ← 0.5		3019.241547 (93)	0.000112
	2 ← 2	1.5 ← 1.5, 2.5 ← 1.5	3019.6542 (1)		0.00007
		2.5 ← 2.5, 1.5 ← 2.5	3019.6580 (1)		−0.00007

## 5 Rotational measurements: *N* = 2 ← 1

**Fig. 2:**
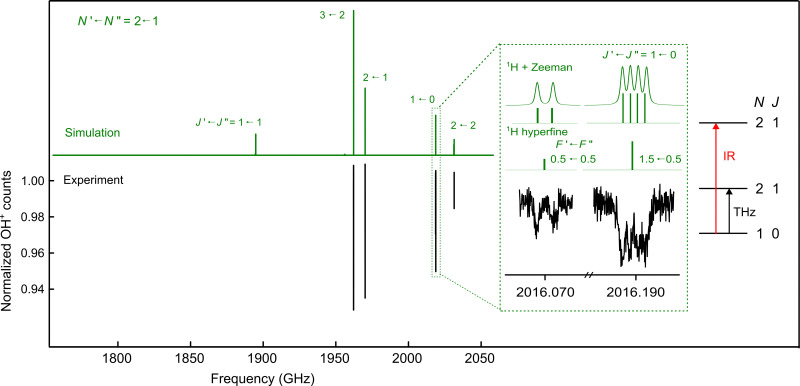
Rotational spectroscopy of the *N* = 2 ← 1 spin manifold of OH^+^ obtained using the DR-LOS “dip” method. The upper panel shows the PGOPHER^71^ simulation (in green), while the lower panel presents the measured spectrum (black). To measure the *J* = 1 ← 0 rotational transition shown in the inset, for example, the IR beam was kept fixed on resonance with the rovibrational (*v*, *N*, *J*) = (1, 2, 1) ← (0, 1, 0) transition at 3019.2375 cm^−1^, while the THz excitation was used to de-populate the (*v*, *N*, *J*) = (0, 1, 0) common level. This results in a decrease of rovibrational LOS signal, *i.e.* in the OH^+^ counts, and the rotational transition is observed as a dip. The inset also provides a detailed illustration of the hyperfine components, along with the effects of Zeeman splitting and Doppler broadening.

The spin manifold of the *N* = 2 ← 1 rotational transition of OH^+^ was predicted to fall exactly in the tuning range of one of our THz sources (1.83–2.07 THz^65^). The lower state quantum levels with *N* = 1 lie at about 30–34.5 cm^−1^, so it was expected that measuring these transitions would be challenging due to the low population of the levels at 4 K. To measure these lines with the IR-THz DR-LOS scheme,^37^ IR transitions connecting to these rotational quantum levels were required, *e.g. N* = 2 ← 1 or *N* = 3 ← 2. As the latter IR transitions were hardly detectable due to the negligible population of the *N* = 2 (≈100 cm^−1^) states at low temperature, we chose the *N* = 2 ← 1 IR manifold shown in Fig. 1 for the double resonance method (or *N* = 0 ← 1). The signal is seen as a drop in the ion counts due to the DR configuration. The measured rotational spectrum is shown in Fig. 2, and the inset shows the applied double resonance scheme.

The spectrum in Fig. 2 is a composite of several individual measurements, in which the IR frequency was kept fixed on the respective resonance, and the submm-wave frequency was scanned back-and-forth multiple times in a given frequency window in fixed steps of 50 kHz. This large step size was necessary because of the extended Zeeman features (see below). The signal in Fig. 2 was normalized following a frequency-switching procedure, where the OH^+^ ion counts monitored in the frequency window of interest are divided by the OH^+^ counts at an off-resonance frequency position. Thus, the baseline in the spectrum of Fig. 2 is close to unity. After normalization, the up-and-down scans were averaged into a single spectrum.

## 6 Rotational measurements: *N* = 1 ← 0

The spin manifold of the *N* = 1 ← 0 rotational transition around 1 THz was targeted with another multiplier chain radiation source (Virginia Diode Inc., VDI). Bekooy *et al.*^1^ pioneered the measurement of these lines 40 years ago, and by revisiting these transitions using our experimental setup at low temperature and low pressure, it was possible to refine the line uncertainties by a factor of about ten. We applied LIICG^55^ as the action spectroscopic method, which is a (sub)mm-wave-only method without the need for a laser. The data of these measurements were recorded employing the COLTRAP machine between December 2016 and January 2017, and are published here for the first time. The measurement procedure and frequency-switching normalization is very similar to that described in the preceding section, and is well documented in the literature.^55–64^

## 7 Extraction of hyperfine line list

The OH^+^ (*X*^3^Σ^−^) radical is susceptible to the Zeeman effect in magnetic fields due to its open shell nature. This was already exploited by the LMR measurements of OH^+^ by Gruebele *et al.*^3^ We observe a splitting of lines in our DR-LOS and LIICG measurements, which could be well reproduced by including the Zeeman effect in our PGOPHER^71^ simulations, as shown in the inset in Fig. 2. The magnetic field best reproducing the DR-LOS observations is on the order of 160 µT and thus about three times larger than the magnitude of the Earth's magnetic field here in Cologne. This value agrees well with recent measurements of other open shell species in our laboratory, *e.g.* CCH^+^ (*X*^3^Π)^73^ or HCN^+^ (*X*^2^Π),^53^ indicating that the observed Zeeman splitting may arise from our experimental setup, such as from magnetic parts of the vacuum chamber or the magnetically levitated turbo-pumps. Interestingly, the magnetic field best reproducing the LIICG measurements from 2016/2017 is about 100 µT, which would favor the turbo-pump theory, as a change of pump type indeed happened in-between.

We simulated the lines with PGOPHER including the Zeeman effect and shifted the simulation in frequency until a good match with the experimental trace had been achieved to extract the positions of the field-free hyperfine components from our measured data. We then obtained the line centers by setting the B-field in the simulation to zero. The hyperfine components obtained this way are listed in Table 2. We estimate these field-free line positions to have an uncertainty on the order of 100–200 kHz.

**Table 2:** Observed frequencies of the *N* = 1 ← 0 and *N* = 2 ← 1 rotational transitions of OH^+^ (in MHz). Uncertainties are given in parentheses

*N*′ ← *N*″	*J*′ ← *J*″	*F*′ ← *F*″	This work	Bekooy *et al.*^1^	obs–calc
1 ← 0	0 ← 1	0.5 ← 0.5	909045.00 (10)	909045.2 (10)	0.044
		0.5 ← 1.5	909158.88 (10)	909158.8 (10)	−0.015
	2 ← 1	2.5 ← 1.5	971804.43 (10)	971803.8 (15)	0.119
		1.5 ← 0.5	971805.47 (10)	971805.3 (15)	0.015
		1.5 ← 1.5	971919.46 (10)	971919.2 (10)	0.065
	1 ← 1	0.5 ← 0.5	1032998.36 (20)	−6.5 (3)^a^	0.081
		1.5 ← 0.5	1033005.11 (10)	1033004.4 (10)	0.047
		0.5 ← 1.5	1033112.25 (20)	−6.8 (4)^a^	0.031
		1.5 ← 1.5	1033119.06 (10)	1033118.6 (10)	0.058
2 ← 1	1 ← 1	c			
	3 ← 2	3.5 ← 2.5	1959561.9 (1)^b^		0.138^b^
		2.5 ← 1.5	1959561.9 (1)^b^		−0.313^b^
	2 ← 1	1.5 ← 1.5	1967532.4 (2)		−0.029
		2.5 ← 1.5	1967536.1 (1)		−0.094
		1.5 ← 0.5	1967539.2 (1)		−0.013
	1 ← 0	0.5 ← 0.5	2016070.9 (2)		−0.119
		1.5 ← 0.5	2016191.6 (2)		−0.147
	2 ← 2	d			

a Bekooy *et al.*^1^ reported frequency intervals between hyperfine transitions.

b peaks merged; obs–calc of merged line is −0.048 MHz.

c Hyperfine lines weak and diluted by Zeeman effect. These components have not been targeted.

d Hyperfine lines weak and diluted by Zeeman effect. Only tentative detection.

## 8 Global fit

The OH^+^ frequency data obtained in the course of the present investigation were combined with the most complete dataset for OH^+^, which was latest reported in the work of Markus *et al.*^7^ This resulting linelist was subjected to a fit to determine the spectroscopic parameters employing Pickett's SPFIT program.^74^ The starting values of the parameters were those from the fit of Markus *et al.*^7^ Briefly, the rovibrational energy levels of a diatomic molecule can be represented by the Dunham expression1
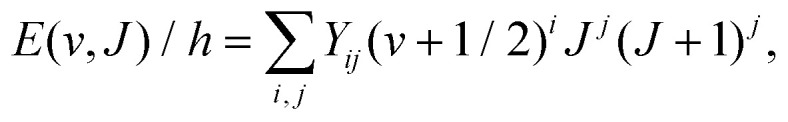
where *Y*_*ij*_ are the Dunham parameters. The electron spin-electron spin coupling parameters *λ*_*ij*_, the electron spin-rotation parameter *γ*_*ij*_ and the isotropic and anisotropic electron spin-nuclear spin coupling parameters *b*_F,*ij*_ and *c*_*ij*_, respectively, can be expanded in an equivalent way. It is worthwhile to mention that in the case of the last two parameters, only fundamental parameters with *i* = *j* = 0 were applied before^7^ and in the present work; vibrational corrections to the HFS parameters could not be determined with sufficient certainty. The resulting spectroscopic parameters are summarized in Table 3, where the values from the fit of Markus *et al.*^7^ are also given for comparison. Overall, the rms error of the present fit (0.904) is slightly smaller than the previously reported value (0.908).

**Table 3:** Global fit Dunham-type spectroscopic parameters^a^ of oxoniumylidene, OH^+^, from the present work in comparison to previous values

Parameter	This work	Markus *et al.*^7^
*Y*_10_ b	3119.2892 (56)	3119.2892 (56)
*Y*_20_ b	−83.1273 (57)	−83.1273 (57)
*Y*_30_ b	1.01953 (241)	1.01953 (241)
*Y*_40_ × 10^3 ^c	2.435 (453)	2.435 (453)
*Y*_50_ × 10^3 ^c	−0.241 (31)	−0.241 (31)
*Y*_01_	503486.85 (22)	503486.90 (26)
*Y*_11_	−22435.77 (62)	−22435.77 (62)
*Y*_21_	308.47 (39)	308.47 (39)
*Y*_31_ c	1.410 (51)	1.410 (51)
*Y*_02_	−58.3435 (29)	−58.3436 (59)
*Y*_12_	1.4529 (33)	1.4523 (37)
*Y*_22_ × 10^3^	7.54 (135)	7.51 (137)
*Y*_03_ × 10^3^	4.118 (20)	4.115 (31)
*Y*_13_ × 10^3^	−0.1359 (155)	−0.1326 (187)
*λ*_00_	64379.4 (25)	64379.5 (26)
*λ*_10_	−253.8 (66)	−254.3 (66)
*λ*_20_	−24.1 (33)	−24.1 (33)
*λ*_01_	−0.781 (24)	−0.68 (11)
*γ*_00_	−4604.96 (25)	−4605.16 (42)
*γ*_10_	143.41 (67)	143.47 (67)
*γ*_20_	−1.42 (35)	−1.45 (35)
*γ*_01_	0.806 (9)	0.796 (17)
*γ*_11_	−0.0385 (76)	−0.0354 (88)
*b*_F,00_(^1^H)	−74.80 (5)	−75.11 (49)
*c*_00_(^1^H)	125.85 (20)	125.95 (87)
*N* d	365	328
rms error^e^	0.904	0.908

a Numbers in parentheses are one standard deviation in units of the least significant digits. All values are in units of MHz unless stated otherwise.

b In units of cm^−1^.

c Sign of *Y*_31_ was incorrect previously.^6,7^

d Number of lines in the fit.

e Root mean square of the errors between the observed and calculated frequencies normalized to their uncertainties.

The line-, parameter- and fit-files of OH^+^ along with auxiliary files will be made available in the data section‡‡https://cdms.astro.uni-koeln.de/classic/predictions/daten/OH+/. of the Cologne Database for Molecular Spectroscopy, CDMS.^6,75^ In addition, the OH^+^*ν* = 0 catalogue file in the entry section§§https://cdms.astro.uni-koeln.de/classic/entries. of the CDMS will be updated and a new entry will be created for the *ν* = 1–0 IR band. For completeness, the spectroscopic parameters for the ground state (*ν* = 0), obtained by a global fit including all field-free measurements involving that state,^1,4,5,7^ are given in Table 4.

**Table 4:** Ground state (*v* = 0) spectroscopic parameters^a^ of oxoniumylidene, OH^+^, from the present work in comparison to values from a previous two-state fit^7^

Parameter	This work	Markus *et al.*^7^
*B*_0_	492346.300 (28)	492346.278 (146)
*D*_0_	57.6232 (45)	57.6166 (52)
*H*_0_	0.004075 (30)	0.004049 (26)
*λ*_0_	64246.45 (7)	64246.00 (55)
*λ*_D0_	−0.77 (3)	−0.54 (12)
*γ*_0_	−4533.60 (4)	−4533.85 (34)
*γ*_D0_	0.7889 (89)	0.7847 (153)
*b*_F_(^1^H)	−74.79 (5)	−75.14 (50)
*c*(^1^H)	125.77 (20)	126.01 (87)

a Numbers in parentheses are one standard deviation in units of the least significant digits. All values are in units of MHz.

## 9 Discussion

The cryogenic and mass-selective ion-trapping approach^50,76,77^ of this work made it possible to refine the *N* = 1 ← 0 transitions of OH^+^ at 1 THz, and to measure the *N* = 2 ← 1 manifold at 2 THz for the first time, utilizing the local oscillator of the upGREAT instrument.^65^ The investigation was enabled by applying different action spectroscopic methods, LIIGC^55,57^ and DR-LOS.^37^ In the latter approach, the rovibrational excitation following the rotational excitation leads to the ions being kicked out of the ion trap and being detected. At higher rotational frequencies, such as the 2 THz regime applied in this work, the rotational photon alone carries enough energy to eject the ions from the trap. This has been demonstrated only very recently and is termed rot-LOS (rotational leak-out spectroscopy^67^). For the strongest transition *N* = 2 ← 1, *J* = 3 ← 2 of OH^+^ at 1959.56 GHz, we tested rot-LOS and indeed obtained a strong signal. Such a rotational-only approach might be useful in the future when no other radiation source is available to perform a double-resonance, or when the rovibrational spectrum is not or only insufficiently known.

Owing to the new data obtained in this work, the uncertainties in the values of several spectroscopic parameters of OH^+^ were reduced by varying degrees, as summarized in Tables 3 and 4. In particular, the uncertainty in the ground-state rotational parameter *B*_0_ decreased from 146 kHz in the previous fit of Markus *et al.*^7^ to 28 kHz in the present work, while the *B*_0_ values themselves remain in excellent agreement, differing by only 22 kHz (Table 4). Although higher-order and purely vibrational parameters were largely unaffected, the uncertainties of *Y*_02_, *Y*_03_, *λ*_01_ and *γ*_01_ have been reduced by factors of ∼1.5 to ∼4 (Table 3). Notable are also the improvements in the HFS parameters *b*_F_ and *c* by factors of about 10 and 4, respectively.

Since OH^+^ is isoelectronic with the NH radical, it is interesting to compare their HFS constants, particularly those of the ^1^H nucleus. In fact, *b*_F_ (H) and *c* (H) of NH were determined as −66.131 ± 0.015 MHz and 90.291 ± 0.084 MHz, respectively,^78^ slightly smaller in magnitude than the corresponding values for OH^+^, which are −74.80 ± 0.05 MHz and 125.85 ± 0.20 MHz (Table 3). These values may also be compared with those of their heavier siblings, PH (−46.543 ± 0.004 MHz and 19.39 ± 0.06 MHz, respectively^79^) and SH^+^ (−56.84 ± 0.03 MHz and 33.48 ± 0.13 MHz, respectively^80^). The HFS parameters of PH and SH^+^ differ not only from those of the lighter OH^+^ and NH counterparts, but also among each other.

With the laboratory measurements of the *N* = 2 ← 1 transition now secured, the question turns to its astrophysical impact. As discussed in the introduction, detections of the *N* = 2 ← 1 transitions were achieved toward three Galactic sources and three galaxies in the local universe, but due to the end of the *Herschel* mission, this observing opportunity no longer exists. However, these lines become redshifted into the observable window in the early universe. Indeed, Riechers *et al.*^30^ show an observed-frame 1 mm spectrum of the *z* = 6.34 starburst HFLS3, obtained with the Z-Spec instrument mounted on the Caltech Submillimeter Observatory (CSO). Based on similar line identifications in Arp 220 at the time (*i.e.*, before the *Herschel*/PACS spectrum was publicly available; González-Alfonso *et al.*^28^), two weak absorption features are identified as excited CH and NH transitions, but both are blended with OH^+^ 2 ← 1 lines at the spectral resolution of Z-Spec. Indeed, recent high-resolution spectroscopy with NOEMA reveals strong contributions of OH^+^*N* = 2 ← 1 absorption to these blended features (D. A. Riechers, priv. comm.). Analogous to the *N* = 1 ← 0 detection in this source, the confirmation of *N* = 2 ← 1 lines opens up the study of excited OH^+^ in the early universe, as is key for the study of the strongest far-IR emitting regions in the circumnuclear regions of the most intense, massive starbursts known.

In conclusion, the investigation of OH^+^ (*X*^3^Σ^−^) in this work using LOS reinforces that this novel method is perfectly suited for investigating open shell cations, as recently also shown for CCH^+^ (*X*^3^Π)^73^ and for the isomers HNC^+^ (*X*^2^Σ) and HCN^+^ (*X*^2^Π).^49,53,81^ While these investigations have primarily focused on rovibrational and rotational spectroscopy, vibronic^82^ and rovibronic^83^ LOS have also recently been demonstrated, highlighting the broad applicability of LOS for investigating molecular cations across a wide range of wavelengths. In this context, revisiting the *A*^3^Π − *X*^3^Σ^−^ electronic spectrum of OH^+^^2,19^ using LOS is a promising future experiment. With the improved determination of the spectroscopic parameters achieved here, searches for rotational transitions of OH^+^ at higher frequencies should also be facilitated.

## Conflicts of interest

There are no conflicts to declare.

## Supplementary Material

CP-028-D6CP00632A-s001

CP-028-D6CP00632A-s002

CP-028-D6CP00632A-s003

## Data Availability

The original data of the IR measurement and the two rotational measurements (at 1 and 2 THz) are made available in the supplementary material as ASCII files together with explanations. In addition, the fit files used (PGOPHER as well as SPFIT/SPCAT) are also provided. Supplementary information (SI) is available. See DOI: https://doi.org/10.1039/d6cp00632a.
